# Advanced Real-Time Monitoring of Rainfall Using Commercial Satellite Broadcasting Service: A Case Study

**DOI:** 10.3390/s21030691

**Published:** 2021-01-20

**Authors:** Gian Luigi Gragnani, Matteo Colli, Emanuele Tavanti, Daniele D. Caviglia

**Affiliations:** 1 Department of Electrical, Electronic, Telecommunications Engineering, and Naval Architecture, University of Genoa, Via All’Opera Pia 11A, 16145 Genova, Italy; m.colli@artys.it (M.C.); emanuele.tavanti@edu.unige.it (E.T.); 2 Artys Srl, Piazza della Vittoria, 9/3, 16121 Genova, Italy

**Keywords:** rainfall monitoring, meteoric flow waters, nowcasting, sensor networks

## Abstract

Correct regulation of meteoric surface and subsurface flow waters is a fundamental goal for the sustainable development of the territories. A new system, aimed at real-time monitoring of the rainfall and of the cumulated rainfall, is introduced and discussed in the present paper. The system implements a Sensor Network based on the IoT paradigm and can cover safety-critical “hot spots” with a relatively small number of sensors, strategically placed, in areas not covered by traditional weather radars and rain gauges, and lowering the costs of deployment and maintenance with respects to these devices. A real application case, based on the implementation of the pilot plant at the Monte Scarpino landfill (Genoa, Italy), is presented and discussed. The system performances are assessed on the basis of comparisons with data provided by a polarimetric weather radar and by a traditional rain gauge.

## 1. Introduction

Nowadays, the risks deriving from extreme weather conditions is constantly increasing and a growing number of people, as well as their available resources, are significantly exposed to flash flood events, all over the planet. Furthermore, the correct regulation of surface and subsurface rain waters must be considered a fundamental objective for the sustainable development of territories subjected to environmental transformation and reclamation processes, such as urban landfills. To cope with these events, it would be highly desirable to have an early warning [1] service that is able to validate meteorological data and promptly alert the civil protection offices, while providing real-time data on the progress of rainfall.

The management of devices and systems for the transfer and collection of rainwater must follow criteria consistent with the principles of permanent site safety. To achieve reclamation, it is necessary to ensure, in particular, the isolation of any contaminating sources as well as the geotechnical stability of the soils and to have efficient environmental monitoring systems. In this sense, the fundamental parameters for the design and management of rainwater regeneration systems are the intensity and duration of rainfall.

At present, the most often used rain sensors are the rain gauge (RG) with the tipping-bucket measuring technique [2] and the weather radar (WR) [3,4]. The first one provides a precise datum at given point-scale locations (how much rain has fallen in the place where it is installed) while the second one provides real data at low resolution (typically of the order of 1 km). The WRs have also significant constraints, concerning both implementation (as they need to be installed in isolated areas with particular features, usually on the top of a mountain) and functional operation (e.g., remote monitoring, shadow areas).

Satellite-based remote sensing techniques based on passive microwave sensors and geosynchronous Earth orbit (geo) infrared (IR) estimates are becoming a relevant alternative in rainfall monitoring since they can provide global coverage and multi-satellite near real-time products. The satellite remote sensing precipitation datasets are made available by national services (e.g., the Integrated Multi-satellitE Retrieval for GPM [5]) and results from combined estimation algorithms maps (such as those developed by NOAA/CPC for CMORPH) provide rainfall maps with a 30-min time resolution on a grid with a spacing of 8 km [6]. However, currently, the usage of such satellite remote sensing products for monitoring convective precipitation events over small-medium sized hydrologic basins and urban environments is still difficult, as finer spatial resolutions and updating time are needed.

To deal with such issue, in recent years, research efforts have been dedicated to the evaluation of the optimal RGs density necessary to retrieve the high spatial variability of the rainfall field [7] through the development and testing of dedicated spatial interpolation techniques such as ordinary Kriging and conditional merging with WR products [8]. Once again, these studies highlighted the need for using high spatial resolution rainfall maps to correctly retrieve the strong variability of convective precipitation events that may occur in limited, and often highly populated, catchments for flash flood risk mitigation [9]. This is particularly necessary in a complex orography composed of small catchments that have to be monitored singularly for flash flood now-casting (it is the case in Mediterranean areas such as southeast France or Alps-Apennines catchments in Italy), in urban drainage waters management or for landfill runoff waters systems (the case showed in our letter) where the use of highly dense RG networks may not be a practicable solution because of its costs of deployment and maintenance.

In this paper, a case study based on the application of an innovative system for rainfall monitoring in limited areas, such as small hydrologic basins and urban environments, is presented. This system (Smart Rainfall System (SRS)) is the result of collaboration about innovation in rainfall monitoring between the start-up Artys and the University of Genoa. By means of proper interpolation techniques, the system allows the real-time reconstruction of the rainfall intensity distribution with spatial and temporal resolutions comparable to or higher than the WR located at Monte Settepani [10], whose data will be used in the following for comparison. Moreover, the SRS has low implementation requirements. The system could allow for early warning and, in principle, also for a now-casting [11,12] service in the monitored area. In this paper, after a short resume of SRS features, the case study involving an urban landfill, located on the hills in the neighborhood of the city of Genoa (Monte Scarpino 44°28′03.9″ N 8°51′17.3″ E), in Italy, is presented and discussed. The system’s performances are assessed on the base of data provided by a polarimetric WR and by a traditional RG located at Monte Gazzo (the nearest one to the test site, at 44°26′48.3″ N 8°49′49.1″ E).

## 2. The Smart Rainfall System (SRS)

Smart Rainfall System (SRS) is an innovative, patented technological solution for estimating and pinpointing rainfall in real-time and providing short-term forecasts of hydrogeological risk [10,13,14,15]. SRS exploits the signal transmitted by commercial geostationary Digital Video Broadcasting Satellites (DVB-S, DVB-S2) to evaluate the quantity of rain falling over the area to be monitored and to provide continuous observation of atmospheric conditions. Suitable sensors have been developed to analyze the intensity of the microwave signal received from common parabolic antennas aligned with selected satellites, mostly operating in the *Ku* band. Each sensor belongs to an infrastructure implementing the Internet of Things (IoT) paradigm. Actually, the whole monitoring system consists of a network of satellite microwave links (SML) realized through the installation of measurement stations (composed by a parabolic antenna and SRS sensor) in the area to be monitored in a potentially extensive and widespread manner; each station continuously send small packets of data to the SRS data center. The latter, hosted on special cloud platforms, analyzes them and derives pluviometric information.

### 2.1. Satellite–Earth Link Model

**Figure 1:**
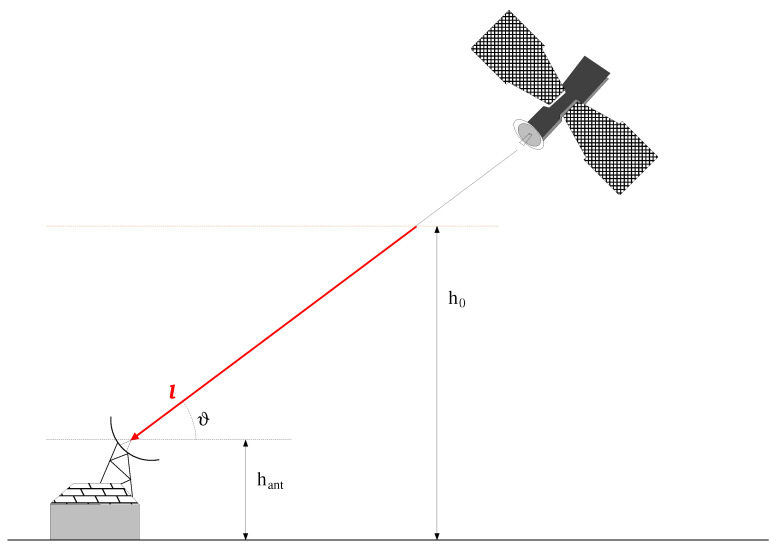
Measurement segment for the rain estimation by evaluation of the DVB-S/S2 signal’s attenuation.

It is well known that rainfall scatters microwaves, and the *Ku* band used for most of DVB-S/S2 transmissions is particularly sensitive to this phenomenon [16,17]. To describe this phenomenon, the radiation emitted by a geostationary satellite can be modeled as a plane wave at the earth station thanks to the far-field condition [18]. Thus, the electric E and the magnetic H fields can be locally described as

(1) $$E\left(r_{rx}\right)=E_{0}\left(r_{rx},r_{tx}\right)e^{-jk\hat{k}\cdot \left(r_{rx}-r_{tx}\right)}\hat{p}$$

(2) $$H\left(r_{rx}\right)=\frac{1}{\eta_{0}}\hat{k}\times E\left(r_{rx}\right)$$

where $r_{rx}$ and $r_{tx}$ are the position vectors for the receiving antenna and the satellite, respectively, $\hat{k}=\left(r_{rx}-r_{tx}\right)/\left|\right|r_{rx}-r_{tx}{\left|\right|}_{2}$ is the unit vector indicating the direction of propagation, $\hat{p}$ is the unit vector for the polarization, $k=\omega \sqrt{\mu_{0}\epsilon_{0}}$ is the vacuum’s wavenumber (with $\mu_{0}$ and $\epsilon_{0}$ magnetic permeability and dielectric permittivity of the vacuum, respectively, and ω=2πf angular frequency), $\eta_{0}=\sqrt{\mu_{0}/\epsilon_{0}}$ is the vacuum’s intrinsic impedance, and $E_{0}$ is the wave’s complex amplitude. The time factor $e^{j\omega t}$ has been omitted to simplify the notation. The power gathered by the antenna can be formulated as [18]

(3) $$P_{R}=A_{e}p_{inc}\left(r_{rx}\right)=e_{p}e_{l}\frac{\lambda_{0}^{2}}{4\pi }G_{rx}p_{inc}\left(r_{rx}\right)=e_{p}e_{l}\frac{\lambda_{0}^{2}}{8\pi \eta_{0}}G_{rx}{\left|E_{0}\left(r_{rx},r_{tx}\right)\right|}^{2}e^{-2\alpha_{rain}l}=P_{R}^{0}e^{-2\alpha_{rain}l}$$

where $A_{e}$ is the antenna’s effective area; $p_{inc}$ is the incident power density; $e_{p}$ and $e_{l}$ are the polarization and load mismatch efficiencies, respectively, $\lambda_{0}$ is the wavelength in the vacuum; $G_{rx}$ is the antenna’s gain; $\alpha_{rain}$ is the rain’s attenuation constant; $P_{R}^{0}$ is the received power in case of clear sky; and *l* is the distance, taken along the wave’s path, between the receiving antenna’s focus and the melting layer, whose altitude $h_{0}$ is here assumed to coincide with the zero-degree isotherm level. The latter is here estimated by means of a linear extrapolation on the base of environmental temperature measurements gathered by two weather stations located at a small geographic distance from the SRS sensors (this approach relies on the assumption of a linear vertical temperature profile). About the quantity *l*, the following relation holds,

(4) $$l=\frac{h_{0}-h_{ant}}{\sin(\vartheta )}$$

where $h_{ant}$ and ϑ are the receiving antenna’s altitude and the elevation angle, respectively. Figure 1 exemplifies the considered configuration. It is important to highlight that $\alpha_{rain}$ explains the rain contribution only; any other attenuation terms can be included in $E_{0}$. Moreover, in writing the Equation (3), it is assumed that the rain’s effect is constant along *l*. Passing to a logarithmic representation, the total attenuation due to the meteorological perturbation is given as

(5) $$L_{rain}^{\left[\mathrm{dB}\right]}=P_{R}^{0,[\mathrm{dBm}]}-P_{R}^{\left[\mathrm{dBm}\right]}=20\alpha_{rain}l\log_{10}\left(e\right)=\gamma_{rain}l^{\left[\mathrm{km}\right]}$$

where $P_{R}^{0,[\mathrm{dBm}]}$ and $P_{R}^{\left[\mathrm{dBm}\right]}$ are $P_{R}^{0}$ and $P_{R}$ expressed in dBm, respectively, $\gamma_{rain}=2\cdot 10^{4}\alpha_{rain}\log_{10}\left(e\right)$ is the rainfall specific attenuation given in dB/km, whereas $l^{\left[\mathrm{km}\right]}$ is *l* expressed in km. Thanks to the semi-empirical model ITU-R P.838-3 [19], the specific attenuation can be related with the rainfall intensity R[mm/h] as

(6) $$\gamma_{rain}=\beta R^{\alpha }$$

The quantities α and β can be computed as follows,

(7) $$\beta =\frac{1}{2}\left[\beta_{H}+\beta_{V}+\left(\beta_{H}-\beta_{V}\right)\cos^{2}\left(\vartheta \right)\cos\left(2\tau \right)\right]$$

(8) $$\alpha =\frac{1}{2\beta }\left[\beta_{H}\alpha_{H}+\beta_{V}\alpha_{V}+\left(\beta_{H}\alpha_{H}-\beta_{V}\alpha_{V}\right)\cos^{2}\left(\vartheta \right)\cos\left(2\tau \right)\right]$$

where τ evaluates to 0, π/2 or π/4 when the polarization is horizontal, vertical, or circular, respectively. The terms $\beta_{H}$, $\beta_{V}$, $\alpha_{H}$, and $\alpha_{V}$ are frequency-dependent; their values can be found in [19] for the working frequency f=12 GHz considered in the following. Given a measurement of $\gamma_{rain}$, the relation in Equation (6) can be inverted to get an estimate of R. However, if higher local accuracy is needed, more sophisticated models can also be adopted (see, e.g., in [20,21,22,23,24]).

### 2.2. The RF Front-End

Summarizing, the system is composed of a network of microwave sensors, each of which measures the signal intensity received from a satellite thanks to a parabolic dish antenna and an Low Noise Block (LNB) converter [25].

**Figure 2:**
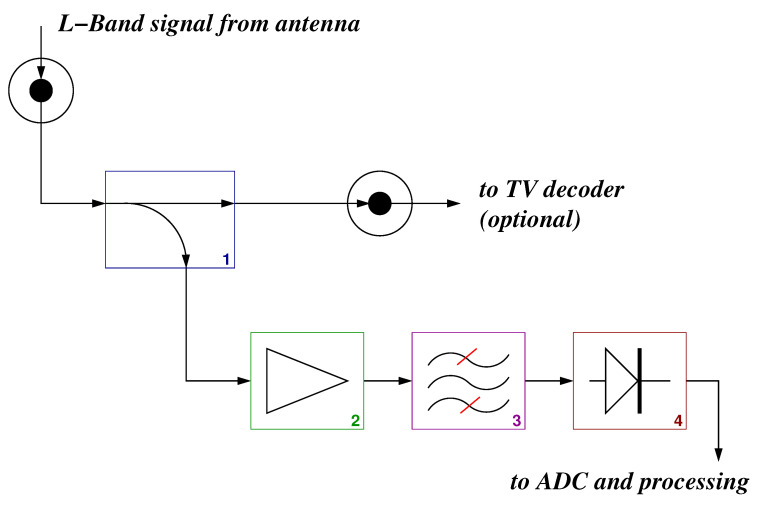
Schematic diagram of the RF front-end of a Smart Rainfall System (SRS) sensor.

In particular, the RF part of a sensor can be schematized according to the block diagram shown in Figure 2. Each sensor receives the signal from a commercial antenna, already converted to *L* band by the LNB, and operates through four fundamental devices:

1. directional coupler,
2. *L*-band Low Noise Amplifier (LNA),
3. *L*-band band pass filter, and
4. logarithmic power detector.

The directional coupler is used to spill a calibrated portion of the received power for the subsequent processing, while most of the signal can still be used in a TV decoder, so that the system can also be used at home as an opportunistic measurement station. The signal portion obtained by the coupler is then amplified by a low-noise amplifier (in the present implementation, an Avago MGA-86563), duly filtered and the resulting power is transformed in a DC signal by a detector having a logarithmic characteristic (Analog Devices AD8314). The DC signal is then converted into digital representation and processed “on-board” by means of a low-cost low-power micro-controller unit (Microchip PIC18F14K22 (MCU)), which generates raw data and provide local input–output as well as access to the internet, through a proper network interface.

### 2.3. High Level Post-Processing

In order to provide more comprehensive information, the SRS system can incorporate measurements taken by traditional sensors (RGs, water gauges, anemometers, CCTV cameras, infrared systems, etc.), making the most of existing investments.

Based on the information analyzed, SRS creates real-time interactive and high-resolution rainfall maps (resolution of the order of 100 m can be expected) of the entire monitored area following the methodology described in Colli et al. [10] and makes them available to its users, via an online service.

SRS is currently provided as a tool for Decision Support System (DSS) for hydro-meteorological risks mitigation, allowing for faster and more efficient hazard analysis compared to traditional monitoring techniques. Decision-makers can predict the consequences of rainfall by monitoring whether critical rainfall levels have been exceeded in the catchment and applying hydrologic/hydraulic models for short-term runoff forecasting (e.g., by adopting semi-distributed or distributed continuous hydrologic algorithms).

SRS also stands out for its low implementation requirements: as a matter of fact, SRS measurement stations use already active infrastructures (satellite telecommunications, internet, and mobile networks) and are made up of off-the-shelf, low-cost components. In addition, each SRS sensor has been designed to optimize power consumption: a set of four sensors co-located can be operated autonomously by a 50 W solar panel with proper battery storage.

For SRS, the Italian patent was recognized in 2014 [26], and it has been extended at the European level in 2019 [27].

## 3. The Monte Scarpino Landfill and the SRS Test-Bed

**Figure 3:**
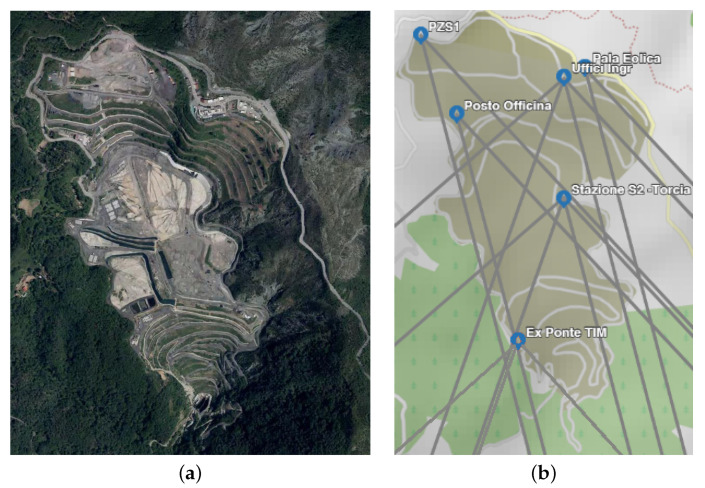
(**a**) Orthophotograph of the Monte Scarpino landfill [28]. (**b**) Map of the geographical locations of the SRS stations in the Monte Scarpino landfill and of the related satellite connection tracks.

The case of the Monte Scarpino landfill in Genoa is of particular importance in relation to the monitoring of areas subject to environmental risk protection. The solid urban waste disposal plant covers an area of about half a million square meters (Figure 3a). Established in 1968, the landfill is located on the heights of Sestri Ponente, in the Metropolitan City of Genoa. The waste disposal plant is about 600 m above sea level and is composed of two modules that today are no longer in operation as the maximum allowed volume for filling has been reached. The over 10 million cubic meters of waste disposed of in the landfill continue to feed a biogas transformation system for the production of electricity (on average 60 million kWh per year entered into the national grid). The Liguria Region and the Metropolitan City of Genoa required AMIU (Azienda Multiservizi e d’Igiene Urbana), the company that operates the landfill, to increase and improve its monitoring through different environmental matrices that influence incoming and outgoing flows of matter and energy.

In this context, particular attention has been paid to the problem of water pollution [29,30]. In the event of sudden, intense and highly localized precipitation phenomena, it is necessary to measure rainfall in real time, identifying the “hot spots” in which the events occur. There is a distance of 1.3 km and an elevation of about 300 m between the entrance to the landfill, to the north, and the borders to the south of it, where the containment tanks of the percolate are located [29]. The levels in the tank and the inflows must be constantly monitored during the rains. A system that acquires data in real time can also ensure greater safety for operators as well as it can generally allow for the activation of emergency procedures based on nowcasting [11,12].

### 3.1. The SRS Test Bed

The planned network will be eventually composed of 18 sensors distributed in 6 different sites enclosed in the landfill’s perimeter. This would allow for an estimated coverage of 9.8 km^2^, providing the capability to monitor the evolution of the rainfall also on areas adjacent to the landfill. In Figure 3b, a map of the geographical location of the SRS stations and of the satellite connection tracks is shown. At present, only eight sensors are fully functional. Table 1 summarizes the set of sensors adopted for the analysis in the following, with their population of satellites.

In Table 1, the name “Turksat 42E” refers to a constellation of two satellites (namely, Turksat 3A and Turksat 4A), co-located at the 42° East orbital position in the Clarke Belt, operated by the Türksat A.Ş. company (Gölbaşı, Turkey) [31]. In the same Table, “Astra 19.2E” refers to the constellation of Astra communications satellites co-located at the 19.2° East orbital position [32], owned and operated by SES S.A., based in Betzdorf, Luxembourg. At present such group consists of four satellites, namely, Astra 1KR, Astra 1L, Astra 1M, and Astra 1N. Both constellations broadcast plenty of channels in the Ku band on strong beams directed towards Europe.

**Figure 4:**
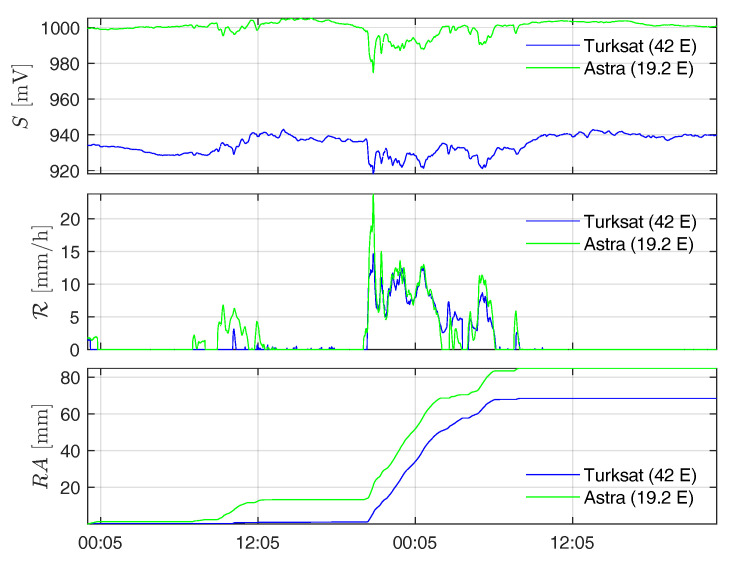
Time series of signal strengths *S* $\left[\mathrm{mV}\right]$ (**top panel**), rainfall intensity R [mm/h] per minute (**middle panel**), and accumulated rainfall *RA* [mm] (**bottom panel**) measured by the SRS sensors at the “Pala Eolica” site in the days 4 May and 5 May 2019. Blue line: Turksat 42 E. Green line: Astra 19.2 E.

**Figure 5:**
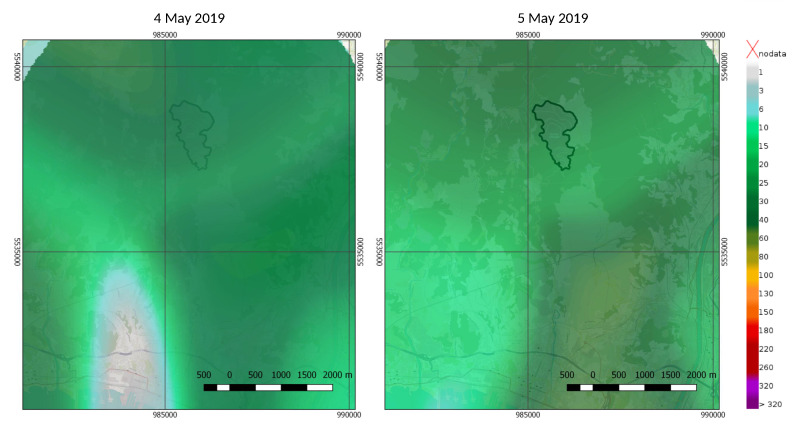
Daily rain accumulation maps produced by the SRS system for the event of 4–5 May 2019, at the Monte Scarpino landfill. The landfill area is highlighted by the black contour. The unit of the colorbar is [mm/h].

To provide an example of the measurements provided by the SRS system we show the behavior of the sensors operating at the “Pala Eolica” site (Table 1) during a typical event (namely, the one occurred on 4–5 May 2019). In Figure 4, the one-minute rainfall intensity, along with the corresponding signal strength and accumulated rain, are reported. The two panels of Figure 5 show instead the spatial distributions of the accumulated daily rainfall for the same rain event.

### 3.2. Reference Rainfall Measurements

If global satellite remote sensing rainfall products are becoming more reliable and accurate as research and development efforts in this field advance, on the other hand, the today state-of-the-art literature suggests the use of RG and WR measurements to perform evaluation analysis of microwave links performances in measuring rain (see, e.g., in [33,34,35]). The motivation is that the RGs are characterized by very low uncertainties [2], especially when compared to remote sensing products [36], and are preferable to perform comparison over small areas [37]. Ground-based WRs represent another valuable standard system for this sort of comparative analysis as their maps provide a representation of rainfall fields with a 1 km spatial resolution and at 10 min time intervals. Even if such features are not fine enough to depict the time-space distribution of highly convective rainfall over small hydrologic basins, like the one considered in the Monte Scarpino landfill use case (torrent Chiaravagna catchment), the radar products constitute a valuable term of comparison for other rainfall map retrieval techniques.

The rainfall measurements considered for analyzing and comparing the SRS results are provided by the Monte Gazzo tipping-bucket RG (characterized by a 0.2 mm rainfall sensitivity) and the Selex-Gematronik GPM250C C-band polarimetric radar, located on Monte Settepani (44°14′45.4″ N 8°11′50.5″ E) in the province of Savona, at a distance of about 57 km from the landfill. The radar and the RG are operated by the Environmental Protection Agency of the Liguria Region (ARPAL), and also by the Italian Department of Civil Protection.

**Figure 6:**
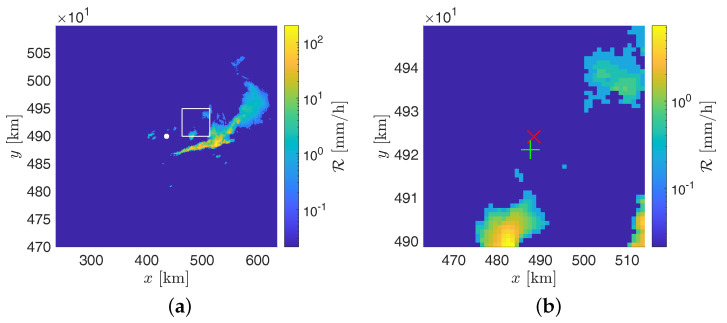
Example of precipitation map (**a**) provided by the GPM250C weather radar on 4 July 2018, at 03:50 UTC. The radar position is indicated by the central white dot. The area containing the Monte Scarpino landfill and the RG at Monte Gazzo is delimited by the white square, whose side length is about 50 km. A zoomed view of this area is provided in subfigure (**b**), where the red cross is centered on the landfill and the green plus sign indicates the RG.

A multiparameter, multirelationship algorithm based on a decision tree is implemented in the Monte Settepani radar system and used for the estimation of rainfall intensity from measurements of polarimetric variables. For interested readers, a detailed description and validation of the algorithm used by the Monte Settepani radar can be found in [38]. The produced precipitation maps have spatial and temporal resolutions of $1\times 1\;{\mathrm{km}}^{2}$ and 10min, respectively. An example of a precipitation map is shown in Figure 6.

To quantitatively evaluate the proposed estimation method against the aforementioned reference approaches, the following error measures are considered:

(9) $$e_{\left\{r,g\right\}}^{in}=E\left(R-R_{\left\{r,g\right\}}|t\in W\right)$$

(10) $$e_{\left\{r,g\right\}}^{out}=E\left(R-R_{\left\{r,g\right\}}|t\notin W\right)$$

where $R_{r}$ and $R_{g}$ are the rain intensities returned by the radar and the RG, respectively; *E* is the expectation operator; and *W* is the time window that strictly contains the rainfall.

### 3.3. Estimation of $h_{0}$

As mentioned above, to estimate the altitude $h_{0}$ of the zero-degree isotherm, SRS performs an extrapolation by assuming a linear vertical profile of the environmental temperature based on the measurements collected by two weather stations located in the neighborhood at two significantly different altitudes. In the specific test case, both of them are operated by ARPAL, and are located respectively at Monte Gazzo (distance from the top of the landfill 3.55 km, direction S (186°), 419 m AMSL) and at Monte Penello (distance 4.29 km, direction E (280°), 980 m AMSL).

## 4. Experimental Results

In this section, in order to test the systems against data provided by other measuring devices, two different events are examined, both pertaining to summer 2018, one in July and the other in August.

In particular, a comparison between the estimates of rainfall intensity returned by the WR described in Section 3.2, a RG located on Monte Gazzo, and the proposed SRS sensors is provided. The radar reference data for the comparisons carried out in the following are obtained by evaluating the estimated rain intensity in the map’s pixel that contains the Monte Scarpino landfill.

### 4.1. Event of 4 July 2018

**Figure 7:**
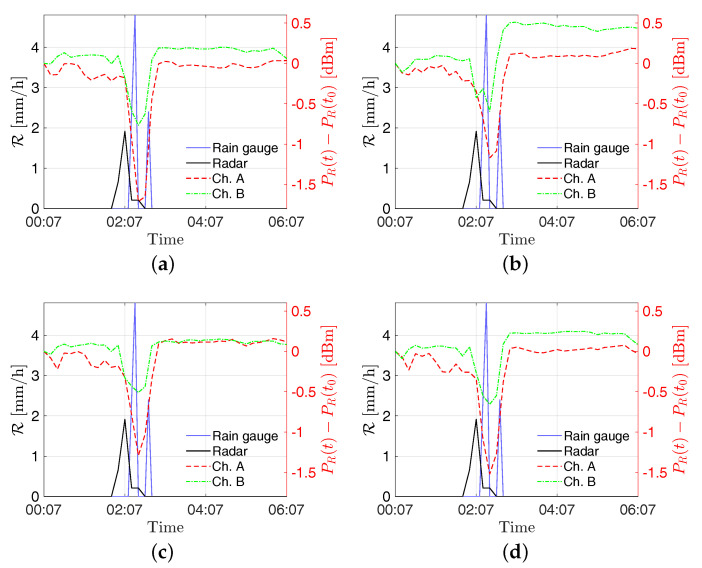
Received power variation $\left[\mathrm{dBm}\right]$ at the sites (**a**) S2—Torcia, (**b**) PZS1, (**c**) Pala Eolica, and (**d**) Uffici Ingr on 4 July 2018. The signal power variations for channels A and B refer to the right *y*-axis, whereas the rain intensities R provided by the radar and the RG refer to the left *y*-axis.

**Figure 8:**
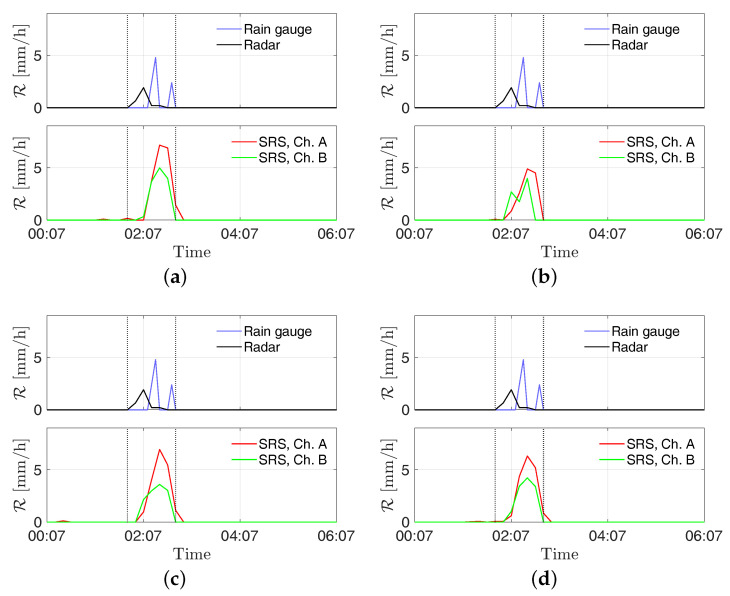
Time series of rainfall intensity R [mm/h] measured at the sites (**a**) S2—Torcia, (**b**) PZS1, (**c**) Pala Eolica, and (**d**) Uffici Ingr on 4 July 2018. The vertical dotted lines delimit the time window *W*.

In Figure 7, the received powers by the stations “S2—Torcia”, “PZS1”, “Ufficio Ingr”, and “Pala Eolica” are shown along with the rainfall intensity estimated from the radar and the RG for the event on 4 July 2018. In order to allow for an easy comparison of data, the SRS measurements are shown as $P_{R}\left(t\right)-P_{R}\left(t_{0}\right)$, where $P_{R}\left(t\right)$ is the received power’s time series and $P_{R}\left(t_{0}\right)$ is the received power at the start of the displayed time window. In Figure 8 the estimates of rainfall intensity returned by the stations “S2—Torcia”, “PZS1”, “Ufficio Ingr”, and “Pala Eolica” are shown along with the ones returned by the radar and the RG for the event on 4 July 2018. Finally, the estimation errors are reported in Table 2.

### 4.2. Event of 14 August 2018

**Figure 9:**
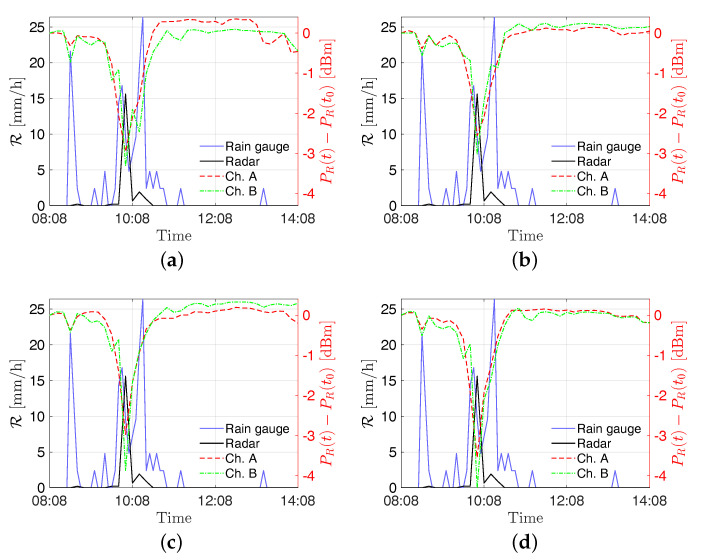
Received power variation $\left[\mathrm{dBm}\right]$ at the sites (**a**) S2—Torcia, (**b**) PZS1, (**c**) Pala Eolica, and (**d**) Uffici Ingr on 14 August 2018. The signal power variations for channels A and B refer to the right *y*-axis, whereas the rain intensities R provided by the radar and the RG refer to the left *y*-axis.

**Figure 10:**
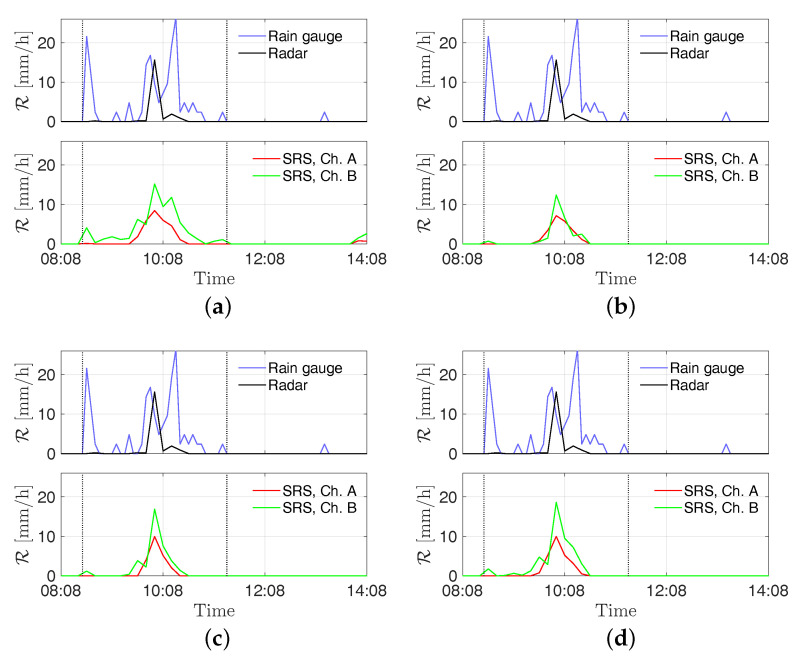
Time series of rainfall intensity R [mm/h] measured at the sites (**a**) S2—Torcia, (**b**) PZS1, (**c**) Pala Eolica, and (**d**) Uffici Ingr on 14 August 2018. The vertical dotted lines delimit the time window *W*.

Results are presented as in the previous Section 4.1: in Figure 9, the received powers by the stations “S2—Torcia”, “PZS1”, “Ufficio Ingr 1”, and “Pala Eolica” are shown along with the rainfall intensity estimated from the radar and the RG for the event on 14 August 2018. In Figure 10, the estimates of rainfall intensity returned by the stations “S2—Torcia”, “PZS1”, “Ufficio Ingr”, and “Pala Eolica” are shown along with the ones returned by the radar and the RG for the event on 14 August 2018. Finally, the estimation errors are reported in Table 3.

### 4.3. Comments

As can be seen in Figure 7 and Figure 9, the rising of the perturbation causes a visible drop in the power received by the SRS stations. This behavior is exploited to derive the estimated rain intensities. According to the results shown in Figure 8 and Figure 10, and the errors reported in Table 2 and Table 3, an overall good level of reliability of the system can be asserted. However, a higher relative accuracy has been achieved for the event on 14 August 2018; the rainfall has been significantly more intense on that date with respect to the 4 July, resulting in a stronger effect on the captured signal.

Some of the differences among the estimates can be explained with the particular features of the involved systems. First of all, the rain intensity returned by the radar is based on an empirical model that aims to estimates the rainfall at the ground level on the base of reflectivity measurements of the atmosphere (in [38] a detailed explanation of the algorithm can be found); this can lead to some differences with the readings provided by the RGs, which collect the rain at the terrain level. Moreover, the SRS stations at the Monte Scarpino landfill are located about 3 km away from the ARPAL one at Monte Gazzo (the SRS system and the ARPAL RG belong to different pixels of the radar precipitation map).

**Figure 11:**
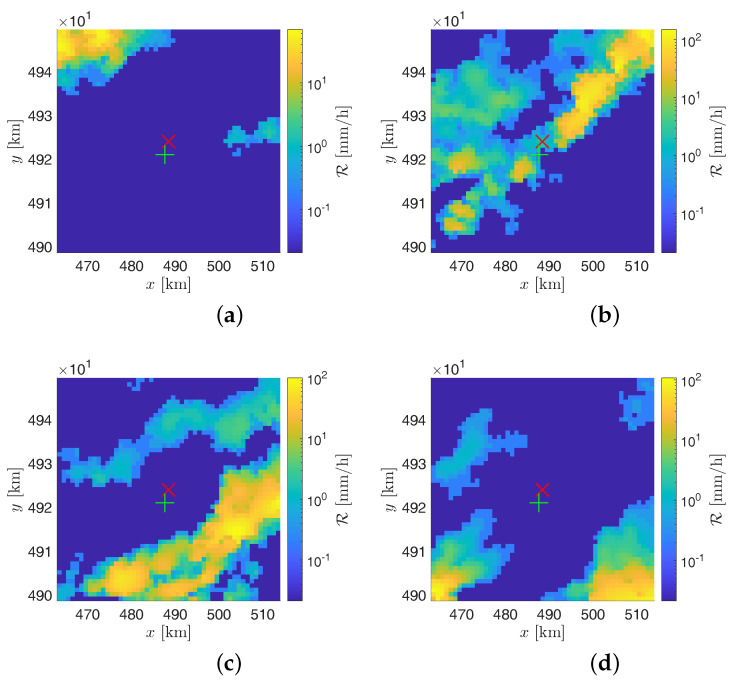
Precipitation maps provided by the GPM250C weather radar on the 4 July 2018 at (**a**) 1:10 UTC, (**b**) 2:00 UTC, (**c**) 2:40 UTC, and (**d**) 3:30 UTC. The red cross indicates the Monte Scarpino landfill, whereas the green plus sign indicates the RG at Monte Gazzo.

A more detailed analysis can be carried out focusing the attention on the evolution of the involved meteorological perturbations. In particular, as for the event of 4 July 2018, a time delay of about 20 min can be noted between the peak of the rain intensity sensed by the polarimetric radar of Monte Settepani and the rain intensity’s peak measured by the sensors network of the SRS. This behavior can be ascribed to the fact that SRS senses the perturbation when it crosses the station–satellite links while the WR measurements are referred to 1 km^2^ tiles of territory. To better understand this point, the rainfall intensity R maps of the WR taken at 1:10 UTC, 2:00 UTC (corresponding to the rain intensity peak according to the WR), 2:40 UTC, and 3:30 UTC are reported in Figure 11a–d for the event on 4 July 2018. It can be noticed that the perturbation is traveling toward the landfill area (represented by the red cross) coming from the north-west direction, whereas, as it can be deduced from Figure 3b, the station–satellite links are located at the south of the landfill. Therefore, the perturbation is not intercepted by the station-satellite links until it arrives in the very proximity of the Scarpino landfill. Such an effect can be effectively mitigated by considering satellite microwave links covering a larger portion of territory than the monitored catchment in order to nowcast precipitation fronts approaching from the north quadrants. Nevertheless, the delay of the rainfall’s peak perceived by the SRS with respect to the RG is only 5 min.

**Figure 12:**
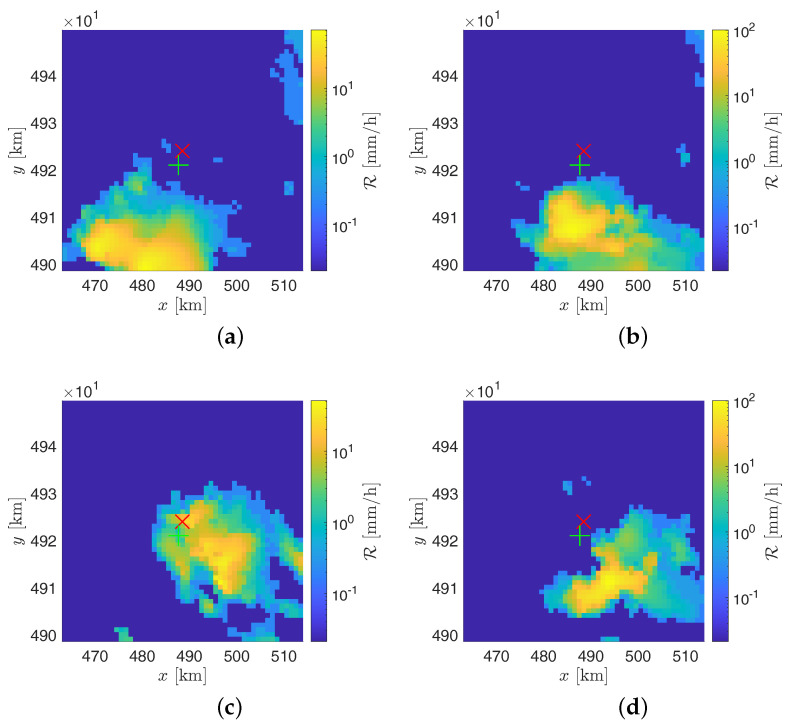
Precipitation maps provided by the GPM250C weather radar on the 14 August 2018 at (**a**) 8:00 UTC, (**b**) 8:50 UTC, (**c**) 9:50 UTC, and (**d**) 10:50 UTC. The red cross indicates the Monte Scarpino landfill, whereas the green plus sign indicates the RG at Monte Gazzo.

As for the event on 14 August 2018, on the other hand, the perturbation was born at the south of the landfill, and so it was detected by SRS with no delay. In Figure 12a–d, the related rainfall intensity R maps generated by the WR, at 8:00 UTC, 8:50 UTC, 9:50 UTC (corresponding to the rain intensity’s peak according to the WR), and 10:50 UTC are shown. About the RG’s data, two strong peaks are present that cannot be found in the WR and SRS’s outputs; these differences are probably due to the distance of 3.55 km existing between the RG and the landfill, which sometimes can lead to significantly different punctual readings.

## 5. Conclusions

In this paper, a new system for real-time monitoring of rainfall and accumulated rainfall has been presented and discussed. The system implements a Sensor Network based on the IoT paradigm and can cover safety-critical “hot-spots” with a relatively small number of strategically placed sensors in areas not covered by traditional weather radars and rain gauges, with the advantage of lower costs of deployment and maintenance.

The Monte Scarpino experimental plant was used as a test-bed for the entire system. The analysis of the sensors performance suggested that a dense measurement grid of satellite microwave link could constitute a valuable complement to traditional monitoring system, especially when the time-space variability of precipitation over small areas has to be monitored in real-time and processed by hazard now-casting tools. The experimental results, obtained by comparing the measurements at four different sites of the plant with those of the WR located at Monte Settepani and a RG placed on the near Monte Gazzo, have confirmed the effectiveness of the system. Therefore, it can be concluded that, although still at an early stage of development, the project is very promising and goes in the right direction to achieve a fast and reliable monitoring system, generally allowing for the activation of emergency procedures based on nowcasting.

About possible future improvements, a study on the exploitation of satellite signals with different polarization is scheduled, in order to gather more information about the meteorological perturbations (e.g., presence of hail).

## Figures and Tables

**Table 1:** Site’s name, reference satellite, antenna’s pointing elevation and altitude, and Ku sub-band for the SRS sensors available at the Monte Scarpino landfill.

*Site*	*Channel A*				*Channel B*			
	*Satellite*	*ϑ* [°]	$h_{0}$ [m]	*Sub-Band*	*Satellite*	*ϑ* [°]	$h_{0}$ [m]	*Sub-Band*
Stazione S2 Torcia	Turksat 42E	29.1	475	High	Astra 19.2E	37.7	475	High
PZS1	Turksat 42E	29.1	560	High	Astra 19.2E	37.7	560	High
Pala Eolica	Turksat 42E	29.1	600	High	Astra 19.2E	37.7	600	High
Uffici Ingr	Turksat 42E	29.1	590	High	Astra 19.2E	37.7	590	High

**Table 2:** Mean errors computed inside and outside the rainfall’s temporal windows achieved with the SRS sensors available at the Monte Scarpino landfill on 4 July 2018.

*Site*	*Channel A*				*Channel B*			
	$e_{r}^{\mathrm{in}}$	$e_{r}^{\mathrm{out}}$	$e_{g}^{\mathrm{in}}$	$e_{g}^{\mathrm{out}}$	$e_{r}^{\mathrm{in}}$	$e_{r}^{\mathrm{out}}$	$e_{g}^{\mathrm{in}}$	$e_{g}^{\mathrm{out}}$
Stazione S2 Torcia	3.08	0.01	2.21	0.01	2.07	0.06	1.40	0.06
PZS1	1.90	0	1.46	0	0.96	0.33	1.31	0.32
Pala Eolica	2.68	0.01	2.09	0.01	1.44	0.09	1.46	0.09
Uffici Ingr	2.61	0.01	1.93	0.01	1.74	0.06	1.38	0.06

**Table 3:** Mean errors computed inside and outside the rainfall’s temporal windows achieved with the SRS sensors available at the Monte Scarpino landfill on 14 August 2018.

*Site*	*Channel A*				*Channel B*			
	$e_{r}^{\mathrm{in}}$	$e_{r}^{\mathrm{out}}$	$e_{g}^{\mathrm{in}}$	$e_{g}^{\mathrm{out}}$	$e_{r}^{\mathrm{in}}$	$e_{r}^{\mathrm{out}}$	$e_{g}^{\mathrm{in}}$	$e_{g}^{\mathrm{out}}$
Stazione S2 Torcia	1.35	0.07	3.78	0.88	2.93	0.21	3.66	0.75
PZS1	1.13	0	3.95	0.44	0.79	0	4.03	0.37
Pala Eolica	0.91	0	4	0.46	1.06	0	4.17	0.41
Uffici Ingr	1.03	0	3.86	0.44	1.80	0	4.06	0.35

## Data Availability

Data available on request. Dataset provided by ARPAL could be subject to formal authorization by the Environmental Protection Agency of the Liguria Region. For further information please contact Dr. Matteo Colli (m.colli@artys.it).

## Abbreviations

The following abbreviations are used in this manuscript:

ARPAL	Agenzia Regionale per la Protezione dell’Ambiente Ligure (Environmental Protection Agency of the Liguria Region)
CCTV	Closed-Circuit Television (Camera)
DVB-S	Digital Video Broadcasting Satellite
DVB-S2	Digital Video Broadcasting Satellite second generation
GIS	Geographic Information System
IoT	Internet of Things
ITU	International Telecommunication Union
LNA	Low Noise Amplifier
LNB	Low Noise Block (down-converter)
SRS	Smart Rainfall System
WR	Weather Radar
